# A Text-Book of the Diseases of the Ear

**Published:** 1891-03

**Authors:** 


*A Text-Book of the Diseases of the Ear.* By Dr. JOSEF GRUBER. London: H. K. LEWIS. 1890.

Drs. Law and Jewell, the translators of this admirable work, have rendered a valuable service to English surgeons, and Bristol may claim to have assisted in the good work, inasmuch as the editor acknowledges that his thanks are due to Dr. Wedmore of Bristol, for many suggestions in the revision of proofs.

No less than 117 pages are devoted to the anatomy of the ear; and although we were inclined at first to resent this, yet on carefully reading the text we were so impressed by the accuracy and freshness of the descriptions, as well as by the excellence of the plates, that we welcomed it as one of the best treatises on the subject.

The remainder of the book is divided into two parts, general and special: the former treating of examination, pathology, and therapeutics, in a general manner; and the latter dealing with the principal morbid affections in detail.

The directions given for passing the Eustachian catheter are very good, and are illustrated by a useful diagram. Five movements are described: First, the introduction through the nostril; second, its advancement as far as the posterior wall of the pharynx; third, its withdrawal to the posterior margin of the hard palate; fourth, a second advance as far as the mouth of the Eustachian tube; and fifth, the rotation and pushing forward of the instrument into the orifice of the tube. This description seems to us as good as any we have seen, and is decidedly easier to follow than the method of Löwenberg which is advocated by most English aural surgeons.

The author is rather severe on Politzer's method of inflating the tympanum, and makes a great deal too much of the objections to its use, such as undue distension of the healthy tympanum in endeavouring to inflate the opposite side, forcing the water with some air into the œsophagus, and rupturing the drum membrane by undue force, &c. The method of inflation advocated for replacing Politzer's method is one invented by the author, and consists in making the patient say one of the following sounds: "Hak," "hik," "hek," "hok," "huk," while the air is blown into the nostril. We have tried this, and have found it distinctly inferior to Politzer's method, even when the patient can be prevailed upon to pronounce such un-English sounds as the above.

Inflammations of the middle ear are divided into exudative and plastic, the former being subdivided into catarrhal, purulent, croupous, and diphtheritic. "Experience leads the author to fix six weeks as the limit of duration of an acute idiopathic catarrh of the middle ear arising in an otherwise healthy individual. With a duration of six months he denominates it as subacute; and if longer still, as chronic."

We are much surprised that in a work of this kind no space should be devoted to the more serious complications arising from middle-ear suppuration, such as cerebral abscess, sinus thrombosis, and suppurative meningitis: all of these are dismissed with one short paragraph, and no account whatever of their symptoms or treatment can be found in the book from beginning to end.

The two lithographic plates in the beginning are the best of the kind we have seen, and give to the student an idea of the membrana tympani in health and disease as vividly as this can be conveyed by any artificial means. We look upon the work as the best in the English language on the subject.

